# Brain Connectivity and Graph Theory Analysis in Alzheimer’s and Parkinson’s Disease: The Contribution of Electrophysiological Techniques

**DOI:** 10.3390/brainsci12030402

**Published:** 2022-03-18

**Authors:** Francesca Miraglia, Fabrizio Vecchio, Chiara Pappalettera, Lorenzo Nucci, Maria Cotelli, Elda Judica, Florinda Ferreri, Paolo Maria Rossini

**Affiliations:** 1 Brain Connectivity Laboratory, Department of Neuroscience and Neurorehabilitation, IRCCS San Raffaele Roma, 00163 Rome, Italy; fabrizio.vecchio@uniecampus.it (F.V.); pappaletterachiara@gmail.com (C.P.); lorenzo.nucci.ln@gmail.com (L.N.); paolomaria.rossini@sanraffaele.it (P.M.R.); 2 Department of Theoretical and Applied Sciences, eCampus University, 22060 Como, Italy; 3 Neuropsychology Unit, IRCCS Istituto Centro San Giovanni di Dio Fatebenefratelli, 25125 Brescia, Italy; mcotelli@fatebenefratelli.eu; 4 Department of Neurorehabilitation Sciences, Casa Cura Policlinico, 20144 Milan, Italy; e.judica@ccppdezza.it; 5 Unit of Neurology, Unit of Clinical Neurophysiology and Study Center of Neurodegeneration (CESNE), Department of Neuroscience, University of Padua, 35122 Padua, Italy; florinda.ferreri@unipd.it; 6 Department of Clinical Neurophysiology, Kuopio University Hospital, University of Eastern Finland, 70210 Kuopio, Finland

**Keywords:** EEG, MEG, graph theory, Alzheimer, Parkinson

## Abstract

In recent years, applications of the network science to electrophysiological data have increased as electrophysiological techniques are not only relatively low cost, largely available on the territory and non-invasive, but also potential tools for large population screening. One of the emergent methods for the study of functional connectivity in electrophysiological recordings is graph theory: it allows to describe the brain through a mathematic model, the graph, and provides a simple representation of a complex system. As Alzheimer’s and Parkinson’s disease are associated with synaptic disruptions and changes in the strength of functional connectivity, they can be well described by functional connectivity analysis computed via graph theory. The aim of the present review is to provide an overview of the most recent applications of the graph theory to electrophysiological data in the two by far most frequent neurodegenerative disorders, Alzheimer’s and Parkinson’s diseases.

## 1. Introduction

The brain is one of the most complex and less explored systems of the human body. It consists of 100 billions of neurons that reciprocally communicate through networks of connections. In order to explain the mechanisms of brain networks, the “brain connectivity analysis” was created recently. Theoretically speaking, the analysis consists of three main types of connectivity: structural, functional and effective connectivity. Structural connectivity is based on anatomical constraints, that is, the set of physical (fibers) and structural (synaptic) connections linking neuronal units at a given time. Anatomical connectivity refers to a network of synaptic connections linking sets of neurons or neuronal elements, as well as their associated structural biophysical attributes condensed in parameters, such as synaptic strength or effectiveness [1,2]. The “functional connectivity space” is defined as the physical substrate in which all neural information processes happen, thus providing plausible biological boundaries for theories and inferences about neural interactions when analyzing functional neuroimaging data and developing computer simulations. In fact, because the structural/anatomical input/output connections of a given brain region are the main constraints for its functional properties, structural brain connectivity does not rigidly determine neural interactions, but acts instead as a dynamic support that reduces the dimensionality of the neural network state space. Meanwhile, functional interactions contribute to modify the underlying structural substrate by modifying the synaptic connections (i.e., enlarging/reducing the synaptic knob area, forming new synapses, and pruning preexisting synapses) [3].

In fact, functional connectivity is time dependent and captures the patterns of deviations between distributed and often spatially remote neuronal units, measuring the statistical correlation or their time-dependent activity. Effective connectivity describes the set of causal/modulating effects of one neural assembly activity over another and defines their inner hierarchy. Structural connectivity has been usually assessed by high spatial resolution technologies, such as magnetic resonance imaging (MRI-tractography); functional and effective connectivity are largely dependent on calculating the correspondence of neural signals over time, using electrophysiological techniques, such as EEG, TMS-EEG and MEG, that have an excellent temporal resolution and are optimal for calculating such connectivity [4,5]. Moreover, EEG recordings can be carried out in more ecological conditions since they do not need any specific environment, in opposition to the need of a shielded room for the fMRI and MEG recordings.

In the human brain, the connectome concept strongly relies on the evidence that neuronal populations interact with each other by means of their connections as well as of their temporally related dynamics. This is particularly evident when considering the innumerable brain dynamic states, which vary instantly and continuously because of changing sensory inputs from internal and external environments [2]. According to the principles of segregation and integration [6] in the human nervous systems, brain anatomical connections are both specific and variable. Specificity depends on the arrangement of individual synaptic connections between morphologically and physiologically different neuronal types, in the organization of axonal arborizations and long-range connectivity between separate cell nuclei or brain regions [1,2].

Recently, the study of brain connectivity was investigated in two of the main neurodegenerative diseases, in particular Alzheimer’s (AD) and Parkinson’s (PD) disease. In fact, AD is histopathologically defined by the presence of amyloid-beta plaques and tau-related neurofibrillary tangles, which have been associated with local synaptic disruptions, loss of fibers and neuronal death: this evidence suggests that AD is a dysconnectivity disease [7,8,9,10,11,12] whose early stages are due to synaptic failure. In addition, previous studies on PD have shown changes in the strength of functional connectivity between distributed brain regions associated with clinical symptoms, such as motor features [13,14,15], as well as a variety of non-motor disturbances, including cognitive impairment [16].

As already mentioned, one of the emergent tools for the study of functional connectivity is graph theory, which allows describing the brain through a mathematic model, the graph, which provides a simple representation of a complex system. The origins of graph theory and network science are to be found in the distant past, but their application in neuroscience is definitely more recent [17]. With the graph model, the brain is shaped as a network composed by nodes linked by directed or undirected, weighted or unweighted edges [18,19]. The characteristics of the graph are measurable through several parameters; the most explored ones are reported in Table 1.

Within this theoretical framework, the current review provides an overview of the most recent applications of graph theory analysis to electrophysiological data for the study of brain functional connectivity in two of the main neurodegenerative diseases, that is, AD and PD.

## 2. Alzheimer’s Disease and Graph Theory

AD is the most common progressive and multifactorial, neurodegenerative disease in the elderly population and the main cause of cognitive impairment. The histopathological hallmarks of AD are the accumulation of the protein fragment beta-amyloid (plaques) outside neurons and of the protein tau (tangles) inside neurons. These changes are accompanied by the death of neurons and consequently by the damage of brain tissue [28].Over the years, as AD has been increasingly considered as a synaptic disconnection syndrome in its early stages—the pre-symptomatic stage of the disease can last many years and does not manifest due to the “neural reserve” that vicariate the lost functions—its complex brain dynamics have been studied by network approach. In fact, functional brain abnormalities can be reflected in changes of connectivity and networks architecture: this can be useful for the characterization of the brain condition in advance of symptom onset and disease progression. 

Graph theory analysis provides tools to quantify networks properties and to understand the association between various pathological processes at the basis of AD. In recent years, researchers have advanced the idea of interpreting neurophysiological data (from EEG/MEG) via graph theory. For the first time, in 2007, Stam and colleagues applied graph theory methods to the EEG data of AD patients, comparing them to a group of control subjects and using synchronization likelihood (SL) as a measure to weight the graph. The authors demonstrated that the PL measure was higher in AD patients, whereas the C showed no significant alterations between the two groups [29]. The authors concluded that AD patients showed a loss of SW network characteristics, indicating less complexity and organization of the brain. 

Since then, numerous scientists have explored the modulation of both local and global connectivity as computed main indexes, such as PL, C and SW, in the M/EEG frequency bands and over the years, and various reviews have been produced [30,31,32]. More recently, the distinctive features of physiological and pathological brain aging [33] were explored in order to describe the modulation of graph theory parameters in AD compared to healthy elderly people as well as to mild cognitive impairment (MCI) subjects. Indeed, MCI subjects do not yet meet the diagnosis of dementia, but carry a remarkably high risk, since about half of them become demented during a 3 to 5 years follow-up. Furthermore, studies of the graph derived from EEGs of AD patients were increasingly published, thanks to the low cost, large diffusion on the territory and non-invasiveness of the technique. Because of such characteristics, the EEG advanced analysis with graph theory might become a tool for large population screening in the near future [34,35]. In recent reviews, Rossini and collaborators [36] and Hallett and collaborators [37] summarized the results obtained from measures of brain connectivity (including graph theory) and their application in neurological diseases, such as AD, across MRI, EEG and MEG techniques. 

However, some consistent results are available. In general, a decrease in PL was found in the lower alpha [38,39] and gamma bands [38], whereas an increase was found in the theta band [40] and in both the delta and theta bands [41].

Moreover, the C coefficient in AD patients have shown consistently a lower value in the alpha1 and beta bands [38], while a higher value of C was found in the theta and alpha1 bands [40,41], and in the alpha and beta bands [24,41]. 

**Figure 1:**
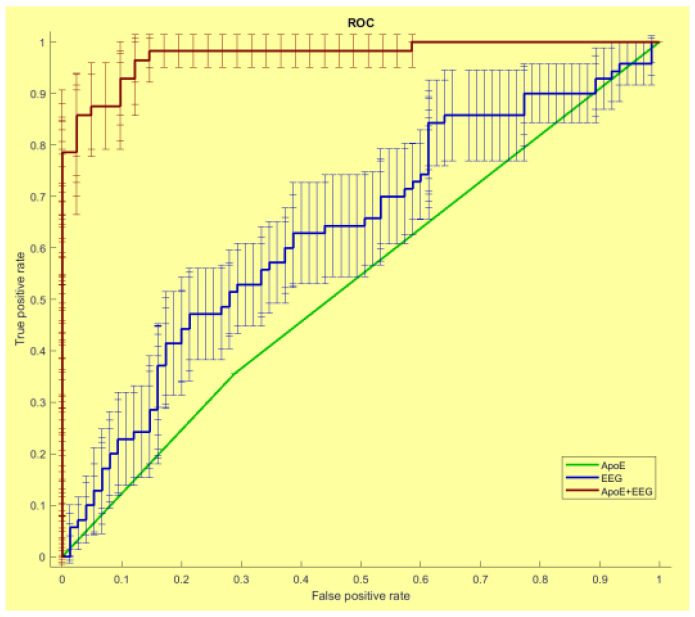
The receiver operating characteristic (ROC) curves illustrating the classification of the Stable and Converted MCI individuals based on the Apo-E (red line), SW (green line) and Apo-E and SW (blue line) values. The area under the (ROC) curve (AUC) was, respectively, 0.52, 0.64 and 0.97. Adapted from [45].

Regarding the SW, the results seem less solid; however, some conclusions can be drawn. Several studies have reported a disruptive reorganization in the brain networks of AD patients in some of the frequency bands analyzed; in particular, the SW values seem to decrease in the delta, theta and in beta bands [33,38,42] and increase in the alpha one [43]. Further studies reported a significant reduction in the SW brain architecture in all the EEG frequency bands computed in mild AD patients compared to healthy controls [25,44]. Other researchers have adopted the SW index as a biomarker of the pathologic conversion of the MCI subject to AD patients, showing a high level of accuracy in combination with other biomarkers, such as Apo-E allele testing [45], as shown in Figure 1. 

A pivotal aspect of graph measures is their potential as a prognostic tool in the conversion to AD status. In this regard, Miraglia and collaborators [46] deepened the analysis of SW in the Default Mode Network in a cohort of MCI subjects, discovering that SW index decreased in the gamma band in converted MCI subjects compared to stable MCI subjects. Moreover, in converted MCI subjects with impairment in linguistic domain, the SW index significantly decreased in the delta band, while in those converted MCI subjects with impairment in the executive domain, the SW index decreased in the delta and gamma bands and increased in the alpha 1 band (Table 1).

**Figure 2:**
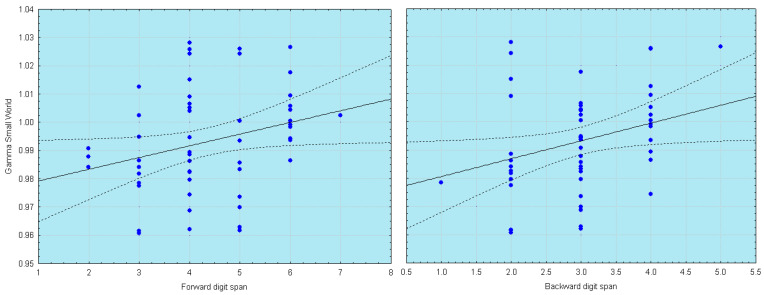
Scatterplots of SW index correlation with memory tasks. Less ordered brain network (as reflected by increasing value of SW) in the gamma band is associated with better memory performance. Adapted from [48].

The most recent studies have intensified the research of the changes of the graph theory’s measures, by analyzing new parameters and correlating them with neuropsychological tests [27,47] (Figure 2) and other biomarkers of AD, such as the hippocampal volume [43]. 

Several research groups are working on this. In a recent study, the number of edges (degree), of inward edges (in-degree), and of outgoing edges (out-degree) were compared among healthy controls, MCI and AD patients with mild dementia by Franciotti and colleagues [27] to evaluate if degree anomaly could involve the measure of degree vertices, called hubs, in both prodromal and over AD stage. Degree, in-degree and out-degree values were smaller in MCI and mild AD than the control group for all hubs, confirming the hypothesis of an affected pattern of information flow in the brain networks. In the same study, the assortative coefficient, a correlation between the degrees of vertices on two opposite ends of an edge, was computed; however, not significant results emerged. Networks with a positive assortative coefficient are resilient high-degree hubs. To the contrary, networks with a negative assortative coefficient are more vulnerable hubs.

Majd Abazid and colleagues [49] used a further innovative approach analyzing a dataset of EEGs of subjective cognitive impairment patients, MCI and AD patients. They quantified the graph links by weighing them by an entropy measure and comparing the accuracy of disease classification to other more used weight measures (i.e., phase lag index and coherence). They demonstrated the higher effectiveness of the entropy measure to analyze the brain network in patients with different stages of cognitive dysfunction.

Furthermore, Kocagoncu and colleagues [50] demonstrated the presence of a correlation between the SW index in the beta and gamma bands and the deposition of the protein tau, meaning that the higher tau burden in early AD’s disease was associated with a shift away from the optimal SW organization and a more fragmented network, especially in the beta and gamma bands. Additionally, several studies have described a link of correlation of the graph parameters with the participant’s generic cognitive status, evaluated by the mini-mental state examination (MMSE) test and memory assessment [27,42,51]. In particular, Franciotti and colleagues [27] found a positive correlation between the clustering coefficient and MMSE inpatients’ groups, namely a higher clustering and higher MMSE, suggesting that high clustering is associated with the robustness of a network and resilience against damage.

**Figure 3:**
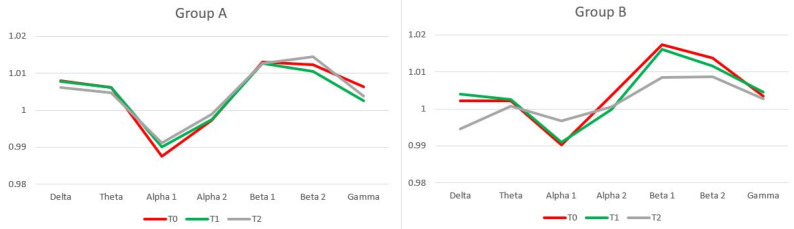
SW evaluation for two AD groups of two type of rehabilitation (repetitive Transcranial Magnetic Stimulation (rTMS) and Cognitive Training (Cog) for Group A and sham rTMS and Cog for Group B) for the evaluation of the effectiveness of the rTMS treatment before (T0), after the rehabilitation (T1) and at the 40 week follow up (T2). Adapted from [52].

Tait and collaborators [51] found a positive correlation between SW calculated in the temporal lobe and the language sub-score of MMSE, indicating that disruption to temporal lobe connectivity plays an important role in the language impairments of AD subjects. In a recent study, the SW measures were used as biomarkers to evaluate the effects of a repetitive transcranial magnetic stimulation and cognitive rehabilitation therapy for AD patients, recording the EEG before and after the treatment [52]. This study showed that the graph parameters can be awarded the role of diagnostic and evaluation biomarkers of AD stages and treatments (Figure 3).

The study of graph theory was applied to explore brain connectivity differences between AD and other dementia as well, such as vascular dementia patients compared to mild cognitive impairment (MCI) and normal elderly (Nold) subjects. It was confirmed that AD patients presented more ordered delta and theta SW organization (lower values), and more random alpha SW (higher values) than Nold and MCI subjects [53].

Finally, Li and collaborators [54] proposed a new combined approach based on an integrative graph analysis, by recording EEG and fNIRS signals in AD and controls subjects during a cognitive task. In particular, the graph indices were calculated from reconstructed EEG sources by using fNIRS localization to assess differences due to the disease. The results revealed lower values of all graph parameters (i.e., degree, C, and centrality) in the alpha and beta bands to the orbitofrontal and parietal regions and across all frequency bands in the frontal pole and medial orbitofrontal frequency, and higher values for the superior temporal sulcus. These findings suggest that the integration of EEG-fNIRS in the graph approaches could be useful to understand the spatiotemporal dynamics of brain activity better.

The main results just described are summarized in Table 2. Clearly, much work has been performed in the description of AD brain characteristics and network architecture, but there is still a lot to achieve in the definition of consensus biomarkers able to intercept the disease at its early stage, as this could provide treatment and rehabilitation strategies to improve the clinical condition of the patients. 

## 3. Parkinson’s Disease and Graph Theory

**Figure 4:**
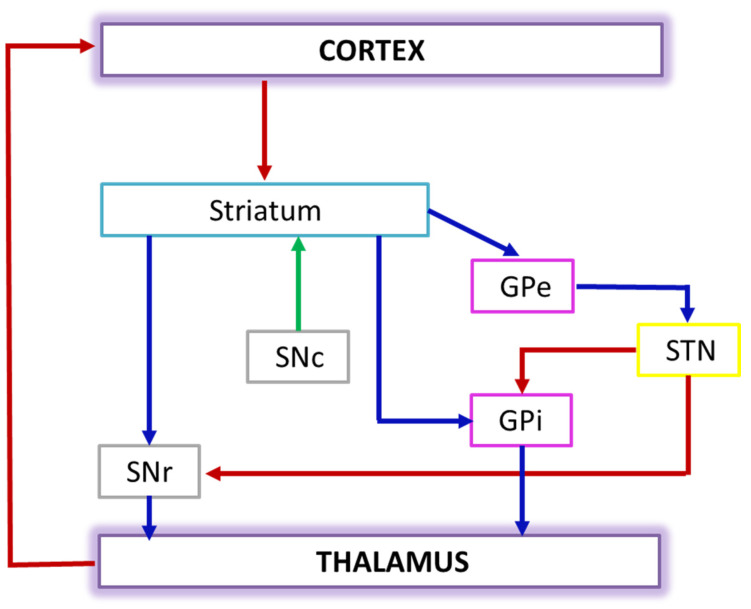
Dopaminergic pathways linking the basal ganglia and the cortex. Connectivity diagram showing excitatory glutamatergic pathways as red, inhibitory GABAergic pathways as blue, and modulatory dopaminergic as green. Their final functional output is the modulation of the cortical activity, mainly for motor-related circuits. Abbreviations: STN: subthalamic nucleus; SNr: substantia nigra pars reticulata; SNc: substantia nigra pars compacta; GPe: external segment of the globus pallidus; GPi: internal segment of the globus pallidus.

PD is a neurodegenerative disease mainly characterized by movement impairment. Non-motor disturbances, including cognitive impairment, mood changes and dementia, are commonly present during the progression of PD [16]. The mechanisms underlying the development of motor and cognitive disorders are not completely understood. PD disorders were largely studied by functional imaging methods that have shown changes in brain functional connectivity. In spite of the promising findings of some studies [56], there are still few applications of graph theory to the M/EEG data of PD patients, probably because it is still assumed that PD is mainly due to subcortical relays degeneration (namely basal ganglia) that could be not captured by scalp recordings, which are dominated by the cortical EEG activity. However, it should be noted that such subcortical relays are heavily and mutually connected to cortical areas and that the clinical symptoms that characterize the disease mainly stem from the disruption of these connections. Therefore, M/EEG data recordings are suitable tools for PD connectivity studies. Figure 4 shows a representation of these relays, that is, the dopaminergic pathways linking the basal ganglia and the cortex. This picture illustrates the link between the cited subcortical structures and the activity of neurons in the cortex, justifying the use of scalp EEG recordings for the evaluation of brain network modulations due to subcortical network deregulations in PD.

Notably, most of the relatively few studies in this disease have used the C and the PL as network measures, and of those selected, only one adds to them modularity and divergence [22] parameters study, whereas another study considers the SW index [21].

The results reported in this review include the most interesting studies concerning the investigation of graph theory application on the electrophysiological data of cognitively normal PD patients, PD subjects with Mild Cognitive Impairments and PD patients with dementia.

In a MEG study [57], cognitively normal PD subjects were compared to the control group and showed a decreased in the C in the delta band, whereas no differences in the PL compared to healthy subjects were reported, indicating the reduced local integration with preserved global efficiency of the brain network. 

In a more recent EEG study, Suarez and colleagues [58] reported a decrease in the C in different bands from a previous work, theta and beta, and in addition, in the same bands, they also found a decrease in PL, highlighting a reduction in functional segregation and an increase in functional integration in both bands.

**Figure 5:**
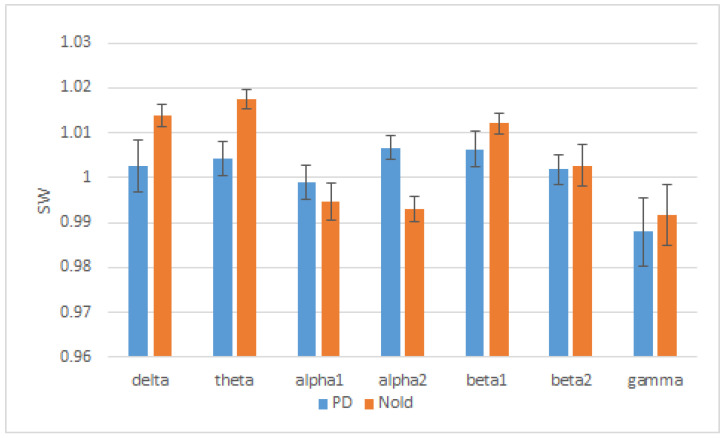
SW index trend in PD patients (blue line) and healthy elderly subjects (red line) in EEG frequency bands (delta, theta, alpha 1, alpha 2, beta 1, beta 2, and gamma). Adapted from [21].

Moreover, in a more recent study on resting state EEG [21], it was found that SW index decreased in the theta and increased in the alpha band, describing a more ordered structure for the lower frequencies and a more random organization for the higher alpha frequencies for PD compared to healthy subjects, as described in Figure 5.

Other studies have followed the temporal development of PD through a follow up or through the assessment of various cognitive impairment stages (MCI and dementia). Over a 4 year period analysis, Olde Dubbelink and colleagues [57] reported, within the same Parkinsonian group, a reduction in the PL in the alpha frequency range and in the C in the theta and alpha bands, showing a progressive impairment in the local integration and an additional loss of global efficiency as reflected in the alpha frequency band along the time.

Regarding Parkinsonian MCI subjects compared to healthy subjects, Suarez and colleagues [58] reported a reduction in the C in the alpha band and a decrement in the PL in the delta and theta bands. Furthermore, a similar reduction in the C was found in the alpha band in the work of Utianski and colleagues [22], who compared MCI PD subjects to PD subjects without cognitive impairment. In a recent study [26], through a dense-EEG source connectivity analysis, it was observed a decreasing tendency of global topological graph features (PL, C, modularity and strength) in the alpha frequency band, from PD patients with normal cognitive profile to PD subjects with dementia. Among functional connectivity studies in PD, the decrease in the patterns in the alpha band seems to be associated with cognitive impairment development. Moreover, the study of Mehraram and colleagues [59] of PD demented subjects showed a decreased C and node degree in the alpha band, an increase in the PL in the alpha band, and of modularity in the theta and alpha bands, compared to healthy subjects, demonstrating that the network measures in the alpha band were significantly affected in demented subjects.

Changes in brain networks in the alpha band can be found even in PD subjects with dementia compared to the ones with a normal cognitive profile. More specifically, a reduction in the C, the PL and of the divergence parameter in the alpha band, and an increase in the modularity index in the alpha and bands were reported [22], demonstrating an impairment of functional connectivity in the background frequency band (alpha). 

Other studies have evaluated graph theory coefficients during the execution of a task. One of them has analyzed the performance of a local contextual processing paradigm [60] in order to demonstrate that functional disconnection is involved with contextual processing deficits in PD subjects. In particular, PD subjects showed a larger C and a longer PL compared to control subjects for predicted targets in the alpha and theta frequency bands, underlining a more structured functional connection in the detection of the predicted target and suggesting that the deficits observed in the process of context in PD may be due to ineffective interactions across cortical regions.

In another study [23], the pre-stimulus network abnormalities of PD patients experiencing pareidolias were investigated during a visual task. A higher global C and parietal efficiency and a decreased frontal degree centrality were found compared to normal PD and healthy subjects in the low-alpha band, suggesting an efficient information transfer within the parietal network and a reduced disengagement of the posterior cortex.

Finally, the latest evidence suggested that graph theory parameters are able to show different modulation of brain rhythms in deep brain stimulation (DBS). Li and colleagues [20] demonstrated that PD subjects with DBS-ON and DBS-OFF reported a lower C and local efficiency in the alpha and beta bands, compared to healthy subjects. Moreover, no evidence was found within patients with PD in DBS-ON and DBS-OFF in any frequency bands. Although significant changes in PL and global efficiency were not found between PD and healthy subjects, the study results indicated decreased brain network segregation in PD subjects and the moving forward to their more random organization, as highlighted in Vecchio and colleagues’ study [21].

Bočkovà and collaborators [61] analyzed graph theory coefficients between normal cognitive PD groups treated by subthalamic brain stimulation (STN-DBS) in OFF and ON stimulation states during a visual task. They found that, in the 1–8 Hz band, subjects responding faster with DBS-OFF demonstrated a decrease in the C and node strength, while the PL was increased in the DBS-ON state compared to DBS-OFF after target stimuli. This network analysis highlights a dysfunction of the large-scale cerebral networks in subjects responding faster with DBS-OFF rather than DBS-ON, reporting global connectivity and slower communication within the frontal network in low frequencies bands (1–8 Hz). These findings suggest that the subjects with such reductions in low frequencies are more likely to develop cognitive deterioration.

The main evidence highlighted in the current review are reported in Table 3. Although only few studies are available on the application of graph theory to the electrophysiological data of PD patients, their results are encouraging for the characterization of the disease and for the response to treatments, suggesting network analysis as a promising tool and interesting field of research to explore.

## 4. Conclusions

The aim of the present review was to provide an overview of the most recent applications of the graph theory to electrophysiological data over two of the main neurodegenerative disorders, that is, AD and PD.

In recent years, the applications of the networks science to electrophysiological data have increased as electrophysiological techniques are low cost, largely available on the territory and non-invasive with the potential to become a tool for large population screening [34,35]. Indeed, health systems are actually looking for a combination f biomarkers characterized by high accuracy, specificity, sensitivity as well as reasonable costs, non-invasiveness, and large availability. We can confidently conclude that the neurophysiological techniques described in this review embody all the required characteristics and are promising as optimal candidates for a first level screening. All of the reported evidence confirms the role of graph analysis as a promising tool in the characterization of the modulation of brain mechanisms of local and global integration in AD and PD as computed by main indexes (such as PL, C and SW in the M/EEG frequency bands) and over the years, and as demonstrated by new parameters (such as assortative coefficient, degree, modularity and divergence). Likewise, the optimal approach for quantifying functional connectivity is an open question [62] still in the absence of methodological convergence. In fact, a graph could be constructed with weighted or unweighted, directed or undirected edges. Moreover, in the case of a weighted graph, there are different weights of the edges [15]. Accordingly, examining the past and more recent studies, the results are sometimes contrasting and clearly dependent on the methods of analysis and on the frequency bands in which the EEG rhythms can be subdivided. The result variability can be therefore explained by the urgent need to share a methodological uniformity in the computation of graph construction and parameters’ computation.

For AD, several studies have reported a disruptive reorganization of the brain networks, suggesting the SW index as biomarker for AD and for the conversion from MCI to AD. Other evidence has reported significant correlations between graph parameters and other biomarkers, such as the neuropsychological test, hippocampal volume, and genetic tests.

Moreover, it is strongly suggested that graph parameters can be awarded the role of diagnostic and evaluation biomarkers of AD and PD stages and rehabilitation treatments, and that it is possible to monitor the temporal development of PD through a follow up over the various cognitive impairment stages.

In conclusion, the graph theory could represent a promising tool for the identification, diagnosis, prognosis and even the identification of rehabilitation treatment for two of the main neurodegenerative diseases, such as AD and PD, to define the effects of the disease, increasing the information provided by traditional topographic mapping. 

However, one of the major challenges of the application of graph theory to electrophysiological data is still to identify the measure that better describes the physiological mechanism under examination based on the data and the experiment. The aim of the current review was to describe the results of the application of graph theory analysis of electromagnetic brain signals in the pathological mechanisms in AD and PD. Several measures were presented, and each of them is more or less significant in the description of different aspects of the brain mechanism, and some of them show a significant correlation to each other. Further studies could be focused on the deeper understanding of the correlations between one measure and another and between the different brain mechanisms involved in the processes of neurodegeneration. This could solve the lack of standardization in methods, thus allowing to successively apply the more significantly measures to specific data and experiments, and to confirm the more promising results on larger populations. Finally, further reviews of the literature should explore the contribution of electrophysiological techniques in other neurodegenerative disorders (i.e., amyotrophic lateral sclerosis, fronto-temporal, vascular, Lewy body types of dementia, etc.).

## Figures and Tables

**Table 1:** Description of the main graph theory parameters.

Parameters	Description
Clustering Coefficient, C	The number of connections that exist between the nearest neighbors of a node as a proportion of maximum number of possible connections. It reflects the tendency of a network to form topologically organized circuits and it is often interpreted as a metric of information segregation in networks [20].
Path Length, PL	The minimum number of edges that must be traversed to go from one node to another. It is used as a measure of global integration of the network [20].
Small-world, SW	The ratio of the normalized clustering coefficient and normalized path length. It describes a balance between segregation and integration network properties integrating the information of global and local network characteristics [21].
Divergence	Measure of the broadness of the weighted degree distribution, where weighted degree is the summed weights of all edges connected to a node [22].
Modularity	Ratio of the intra- and intermodular connectivity strength where modules are subgraphs containing nodes that are more strongly connected to themselves than to other nodes. Modularity is a measure of the strength of the modules [22].
Efficiency	The ability of information exchange within the network [23].
Global efficiency	Measure of network integration and its overall performance for information transferring. This measure is inversely related to the average shortest path length [24].
Local efficiency	Local efficiency, which has a similar interpretation as clustering coefficient, measures the compactness of the subnetwork [25].
Centrality	The importance of a node and its direct impact on adjacent brain areas [23].
Betweenness	Used to investigate the contribution of each node to all other node pairs on the shortest path. It measures not only the importance of the nodes, but also the amount of information flowing through the node [25].
Strength	The sum of weights of connections (edges) of node. The strength can be averaged over the whole network to obtain a global measure of connection weights [26].
Degree	The degree of a node is the sum of its incoming (afferent) and outgoing (efferent) edges [27].
In-degree	Number of afferent connections to the node [27].
Out-degree	Number of efferent connections to the node [27].
Assortativity coefficient	The assortativity coefficient represents a measure of a network’s resilience. It is a correlation coefficient between the degrees of all vertices on two opposite ends of an edge [27].

**Table 2:** Summary of the main results of AD studies reported in the present review.

Authors	Recording Type	Graph Parameters	Main Results (All Results Refer to AD vs. Healthy)
Stam et al., 2007 [29]	EEG	PL C	Beta PL ↑ Beta C ↓ Pearson’s correlation: Beta PL ↑ MMSE ↓
Stam et al., 2009 [39]	MEG	PL C	Alpha 1 PL↓ Alpha 1 C ↓ Pearson’s correlation: Alpha 1 C ↓ MMSE ↓
de Haan et al., 2009 [38]	EEG	PL C	Alpha-1 and beta C ↓ Alpha 1 and gamma PL ↓
Poza et al., 2013 [41]	EEG	PL C	Delta e theta PL ↑ Alpha 2 and beta PL ↓ Delta and theta C ↓ Alpha 2 and beta C ↑
Wang et al., 2014 [25]	EEG	PL C Global Efficiency Local Efficiency SW	PL↑ in all frequency bands (except delta) C ↓ in all frequency bands (except delta) Global Efficiency ↓ in all frequency bands Local Efficiency ↓ in all frequency bands SW ↓ in all frequency bands
Vecchio et al., 2014 [40]	EEG	PL C	Theta PL ↑ Delta, theta and alpha-1 C ↑
Frantzidis et al., 2014 [44]	EEG	SW	SW ↓ Pearson’s correlation: -SW ↓ MMSE ↓; SW ↓ MoCA ↓
Vecchio et al., 2016 [42]	EEG	SW	Pearson’s correlation: Gamma SW ↓ Digit Span Test ↓
Miraglia et al., 2017 [33]	EEG	SW	EO: delta and theta SW Nold>MCI>AD EC: delta SW Nold and MCI > AD
Vecchio et al., 2017 [34]	EEG	SW	Pearson’s correlations: Alpha SW ↓ hippocampal volume ↑ Delta, beta, and gamma SW ↑ hippocampal volume ↑
Saeedeh Afshari and Mahdi Jalili, 2017 [24]	EEG	Global efficiency Local efficiency	Beta global efficiency ↓ Alpha local efficiency↑
Vecchio et al., 2018 [45]	EEG	SW	ROC curve accuracy 97%
Franciotti et al., 2019 [27]	EEG	Degree In-degree Out-degree Assortative Coefficient	Degree, in-degree, out-degree ↓
Li et al., 2019 [54]	EEG	Degree C Centrality	Alpha 2 and beta degree, C, centrality ↓ in orbitofrontal and parietal regions All frequency degree, C, centrality ↓ in frontal pole and medial orbitofrontal regions All frequency degree, C, centrality ↑ in the temporal sulcus
Vecchio et al., 2020 [55]	EEG	SW	ROC curve accuracy 95%
Miraglia et al., 2020 [46]	EEG	SW	Gamma SW ↓ in converted MCI vs. stable MCI Delta SW ↓ in converted MCI in linguistic domain Delta and gamma SW ↓ and alpha 1 SW ↑ in converted MCI in executive domain
Cecchetti et al., 2021 [47]	EEG	PL C	Theta PL ↓ Alpha 2 PL ↑ Theta C ↑ Alpha 2 C ↓
Majd Abazid et al., 2021 [49]	EEG	PL C Degree Efficiency Betweenness	Higher accuracy of classification of AD for the graph parameters
Kocagoncu 2020 [50]	E/MEG	SW	Pearson’s correlation: Beta and gamma SW ↑protein Tau ↑
Tait et al., 2019 [51]	EEG	SW	Pearson’s correlation: Temporal lobe SW ↑ language sub-score ↑
Vecchio et al., 2021 [52]	EEG	SW	Pearson’s correlations: Delta SW ↓ ADAS-Cog ↑ Alpha 1 ↑ ADAS-Cog ↑
Vecchio et al., 2021 [53]	EEG	SW	Delta and theta SW ↓ Alpha 2 SW↑

The arrows refer to an increase (↑) or a decrease (↓) of the indicated parameters in AD patients. All results in the table refer to AD patients compared to elderly healthy controls, except when differently indicated. Abbreviations: EEG: electroencephalography; MEG: magnetoencephalography; PL: path length; C: clustering coefficient; SW: small-world index; MMSE: mini-mental state examination; MoCA: Montreal Cognitive Assessment; EO: eyes open; EC: eyes closed; NOLD: NOrmal eLDerly; MCI: Mild Cognitive Impairment; ROC: received operating characteristics.

**Table 3:** Summary of the main results of PD studies reported in the present review.

Authors	Recording Type	Graph Parameters	Main Results
Fogelson et al., 2013 [60]	EEG	C PL	C ↑ in theta and alpha bands PL ↑ in theta band for PD vs. Healthy subjects.
Olde Dubbelink et al., 2014 [57]	MEG	C PL	C↓ in delta band for PD vs. Healthy subjects C ↓ in theta and alpha 2 bands, PL ↓ in alpha 2 band for PD subjects
Utianski et al., 2016 [22]	EEG	C PL Divergence Modularity	C and PL ↑ in all frequency bands, Divergence ↑ in theta and beta bands and ↓ in delta and alpha bands, Modularity ↑ in all frequency bands, for normally cognitive PD vs. Healthy subjects. C ↓ in alpha 1 band for PD-MCI vs. normally cognitive PD subjects. C, PL and Divergence ↓ in alpha 1, Modularity ↑ in alpha 1 and 2 frequency bands, for demented PD vs. normally cognitive PD subjects.
Hassan et al., 2017 [26]	EEG	C PL Modularity Strength	PL, C, Modularity and Strength ↓ in alpha frequency band for demented PD vs. normally cognitive PD subjects.
Mehraram et al., 2020 [59]	EEG	Node degree C PL SW Modularity	C and Node degree↓ in alpha band, PL ↑ in alpha band and Modularity↑ in theta and alpha bands, for PD demented vs. Healthy subjects. PL ↑ in alpha band, Modularity↑ in theta and alpha bands and SW ↑in theta band, for PD demented vs. AD subjects.
Bočková et al., 2021 [61]	EEG	Node strength C PL Modularity	Node strength ↓, C ↓ and PL ↑ in 1–8 Hz frequencies band for DBS-ON compared to DBS-OFF for subjects responding faster with DBS-OFF rather than DBS-ON.
Suárez et al., 2021 [58]	EEG	C PL Local efficiency Global connectivity	C and PL ↓ in theta and beta bands for normally cognitive PD vs. Healthy subjects. C ↓ in alpha band, PL ↓ in delta and theta bands in PD-MCI vs. Healthy subjects.
Vecchio et al., 2021 [21]	EEG	SW	SW ↓ in theta band and ↑ in alpha 2 band.
Li et al., 2021 [20]	EEG	C PL Global efficiency Local efficiency	C and Local efficiency ↓ in alpha, beta 1 and beta 2 bands for PD subjects in DBS-OFF and DBS-ON vs. healthy subjects.
Revankar et al., 2021 [23]	EEG	C PL Efficiency Centrality	C and parietal Efficiency ↑ in alpha 1 band, frontal Centrality ↓ for PD with pareidolias vs. normal PD and Healthy subjects.

The arrows refer to an increase (↑) or a decrease (↓) of the indicated parameters. Abbreviations: EEG: electroencephalography; MEG: magnetoencephalography; PL: path length; C: clustering coefficient; SW: small-world index; PD-MCI: Parkinson disease with mild cognitive impairments.
